# Supplementary material for: General calibration of microbial growth in microplate readers
Source: Sci Rep. 2016 Dec 13;6:38828. doi: 10.1038/srep38828 (PMC5153849; doi:10.1038/srep38828)
Supplement: Supplementary Information [file srep38828-s1.pdf]

Supplementary Information for "General calibration of microbial growth in microplate readers"

Keiran Stevenson, Alexander F. McVey, Ivan B. N. Clark, Peter S. Swain and Teuta Pilizota

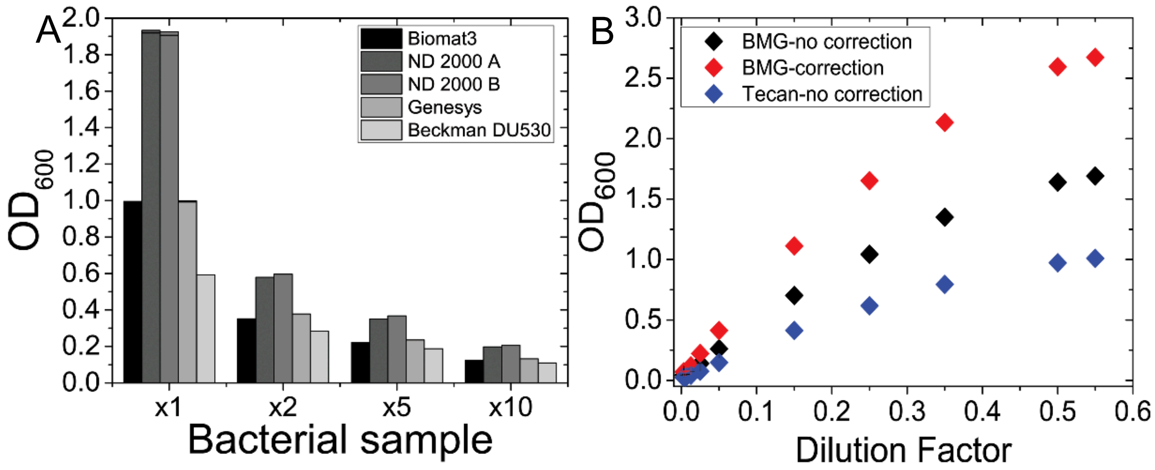

Supplementary Figure 1: (A) Measurements taken of the same sample in five different spectrophotometers. Different spectrophotometers of the same make give the same OD reading, while those produced by different companies measure OD that varies significantly. (B) Calibration performed for spectrophotometers used to obtain growth curves in this work. Calibration was performed using the same plate prepared with a culture of *E. coli* grown to mid-log phase in LB with dilutions as in main text. We report dilution on the x axis to remove uncertainty in determining real concentration. The BMG spectrophotometer provides a path length correction for the Beer-Lambert law (BLL) by default, correcting the OD to that which would be observed by a 1 cm deep well assuming pure absorbance measurements. All *E. coli* measurements were performed in the BMG Spectrostar Omega with path length correction on (red) and yeast measurements were performed in a Tecan Fluorescent spectrophotometer (blue). Conversion factors between the Tecan and BMG were calculated from the data as 1.74 ± 0.02 and 2.75 ± 0.03 for non corrected and corrected readings respectively (*Methods: OD measurements*).

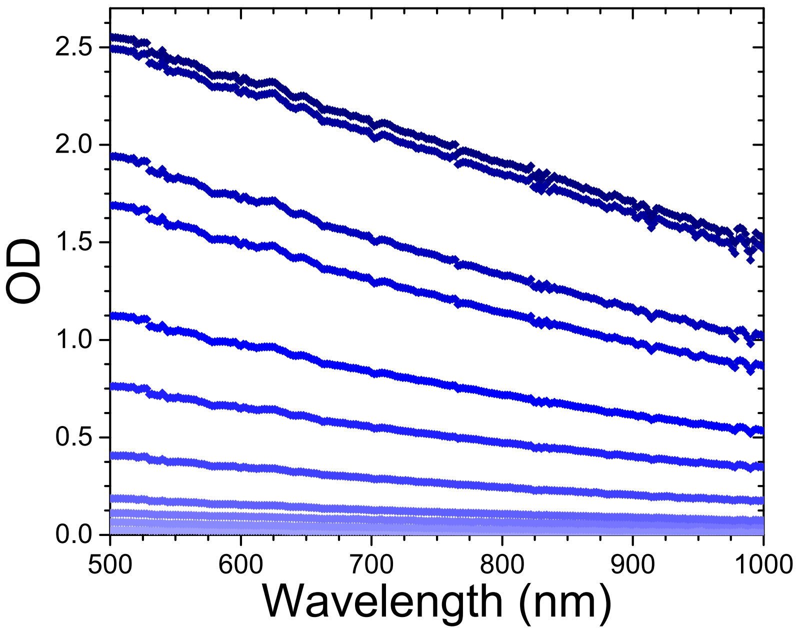

Supplementary Figure 2: OD measurements of *E. coli* samples of different concentrations using different wavelengths. BW25113 Keio collection parent strain was used for this purpose. High concentrations (dark) to low concentrations (light) are indicated with the gradient scale. High concentrations are seen to increase in OD as λ decreases showing the nonlinear dependence of λ on OD. OD measurements on cell expressing fluorescent proteins should include careful choice of wavelength, as absorption caused by the presence of fluorescent proteins can lead to overestimate of cell concentration [1].

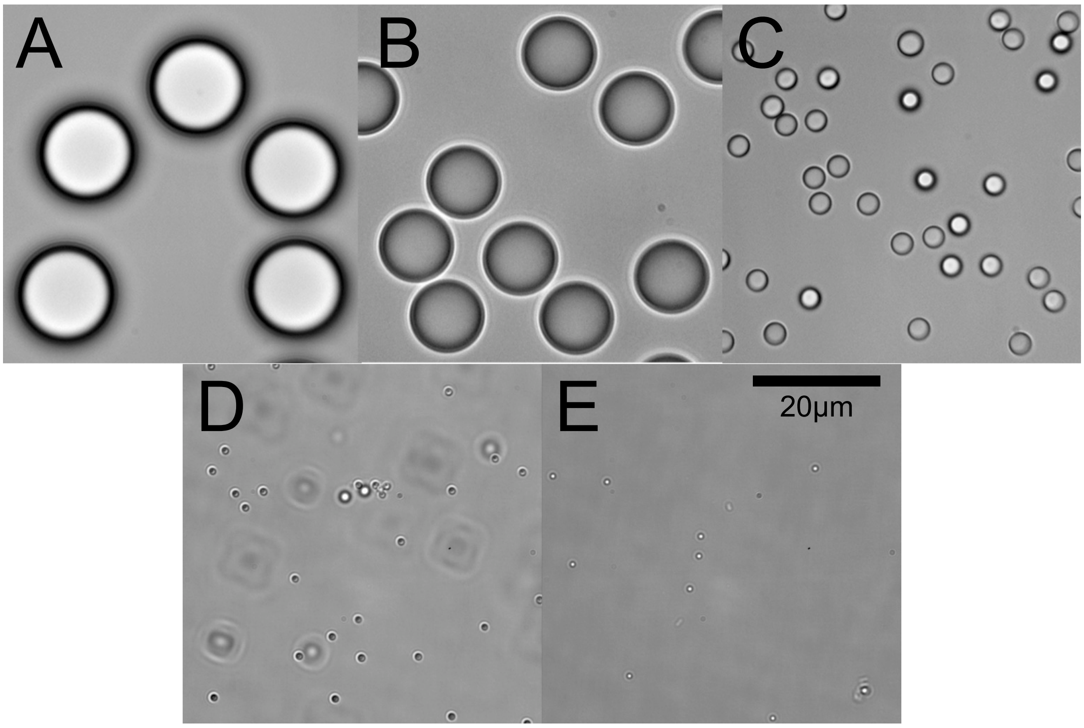

Supplementary Figure 3: Selected brightfield images of beads used in Figure 2. A: 15.7±1.4 µm B: 10.0±0.6 µm C: 3.00±0.07 µm D: 0.96±0.07 µm and E: 0.51±0.01 µm. Scale bar given in E applies to A-E.

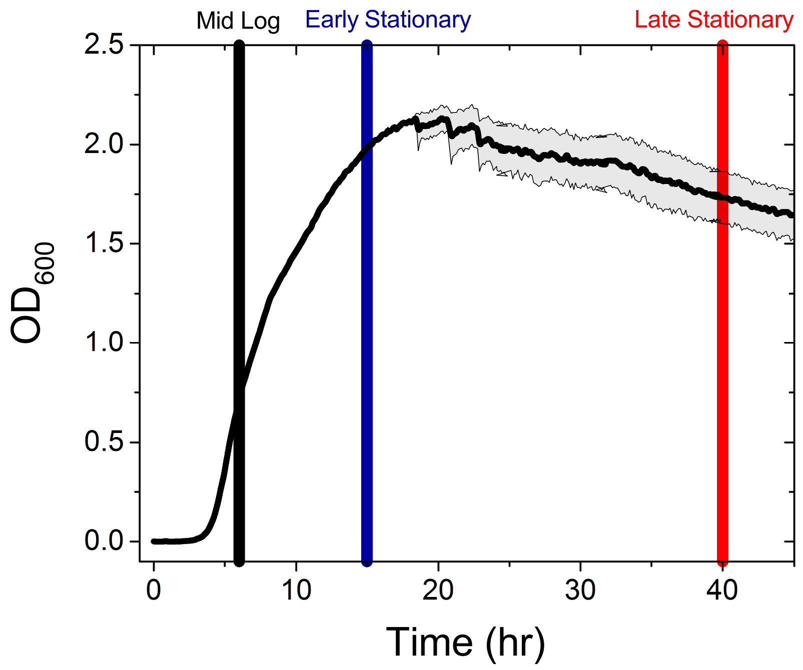

Supplementary Figure 4: Growth curve of *E. coli* sample grown in LB from which the calibration curves for Figure 3 were taken. Mean (black) growth and standard error (grey) was calculated from 10 wells of 300 µl. Cultures of mid log (black), early stationary (blue) and late stationary (red) bacteria were removed at times indicated by the respective vertical lines.

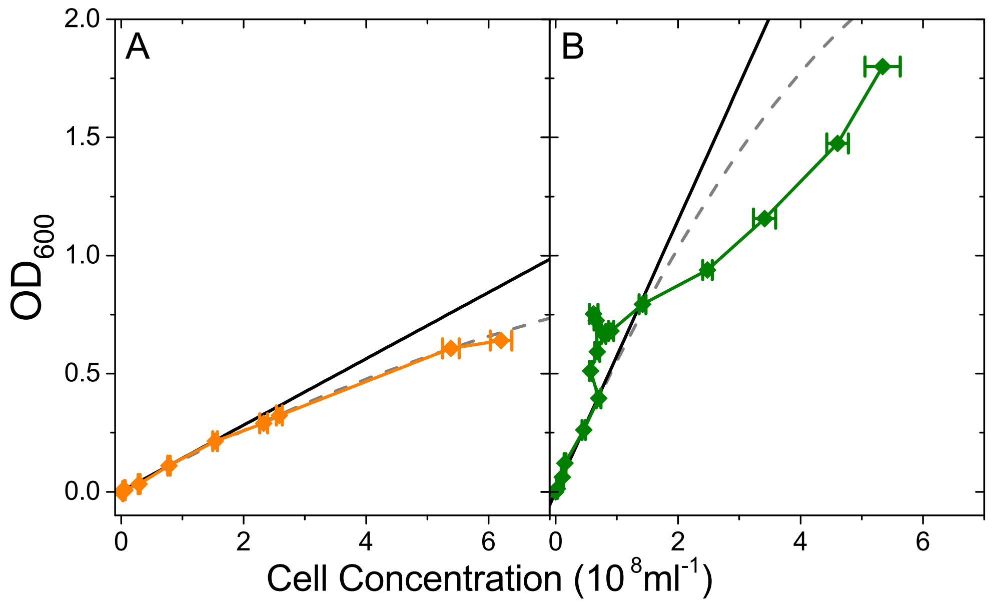

Supplementary Figure 5: C vs OD for growth conditions in Figure 4A and C. Solid and dashed lines represent linear and second degree polynomials fitted to the data (see *Methods* according to the Beer-Lambert Law and scattering theory prediction by Koch [2] respectively. The MM9 (orange) calibration is a parabola as expected [2] (with the exception of the last point obtained when cells started to change size to smaller), however LB+amp (green) calibration shows significant and nontrivial deviation.

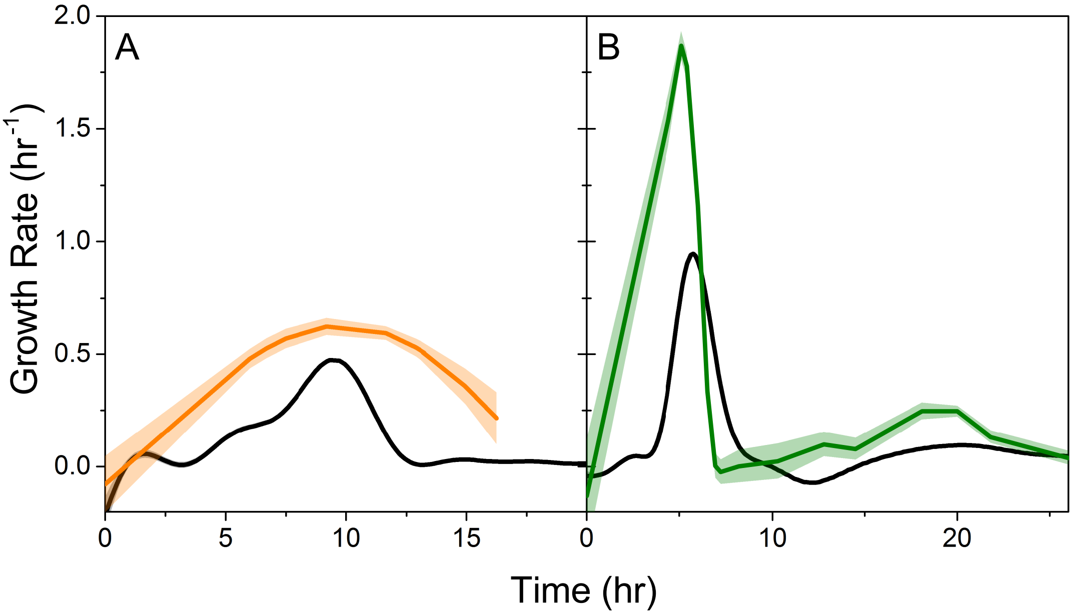

Supplementary Figure 6: Growth rates of *E. coli* were calculated from time derivatives [3] for (A) glucose only media and (B) LB supplemented with 9 µg ml^{-1} Ampicillin. Growth rates are calculated from both the OD_{600} growth curve (black) and the cell concentrations (Orange and Green) presented in Figure 4A and C in the main text. The small difference between maximum growth rate obtained from OD and C seen in (A) results from smaller number of time points obtained for C.

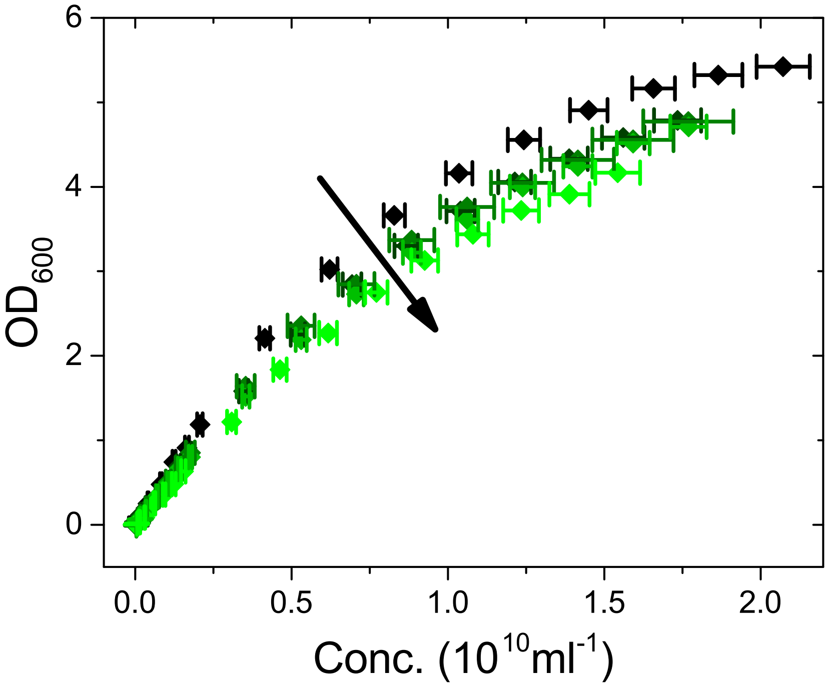

Supplementary Figure 7: Bead concentrations plotted against the resultant OD for 1.0 µm beads in the presence of sucrose. The index of each trace is $n_m = 1.333; 1.339; 1.344; 1.353$ and 1.368 respectively, with increasing brightness of green representing a higher refractive index of media (n_m). Arrow indicates increasing refractive index of media.

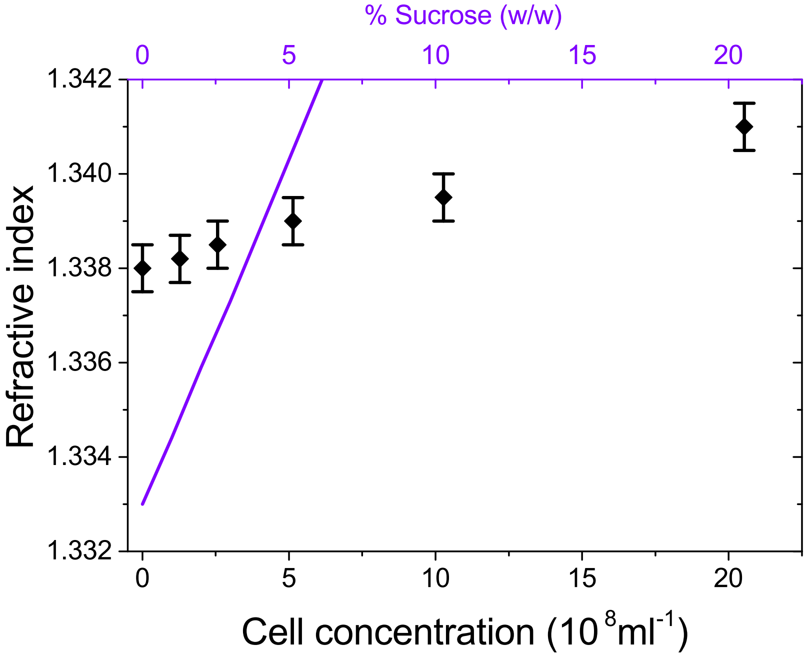

Supplementary Figure 8: Variation in refractive index of LB media as a function of the concentration of lysed *E. coli* cells present. Cell concentrations are measured as outlined in *Methods* before being lysed and added to LB. n_m varies only slightly, even at the highest fractions of intracellular material. In contrast, adding sucrose to ddH$_2$O (purple) produces a far larger variation in n_m.

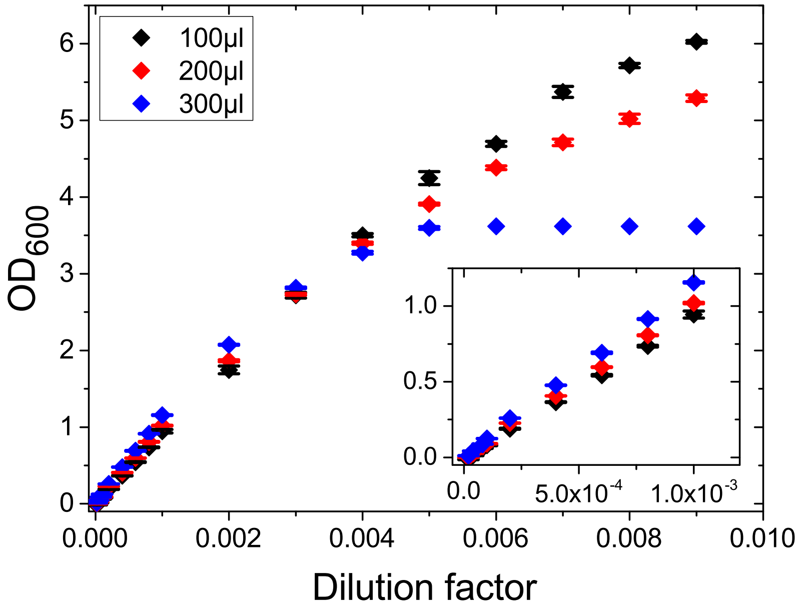

Supplementary Figure 9: OD measurements of 1 µm beads of known concentrations and volumes of 100 µl, 200 µl and 300 µl performed in the BMG Spectrostar spectrophotometer used in this paper. The volume of solution occupies $\frac{1}{3}\times$, $\frac{2}{3}\times$ and $1\times$ the total volume of a single well respectively. The BMG Spectrostar corrects the measured OD using a built in path length correction based on the Beer-Lambert law. However, the measurements for the three volumes do not overlay one another as would be predicted by Beer-Lambert law. The difference between volumes of solution is large for high values of OD, but is also present at OD < 1 as shown in the inset, with the OD of the fully occupied well being higher than those partially filled [4].

Supplementary Table 1: Scattering approximations and their dependency on the radius of the scatterer (r), wavelength of incident light (λ) and refractive indices of the scatterer and the media (n_p and n_m) respectively. n is the relative refractive index between the scatterer and the medium ($\frac{n_p}{n_m}$). Abbreviations: Scatt.: scatterer, sph.: spheres, arb.: arbitrary geometry.

Approx.	Scatt.	Regime	Dependence on N, λ, n and r	
Rayleigh	sph.	$r \ll \lambda$; $\frac{n_p}{n_m} r \ll \lambda$	$\left(\frac{n_p}{n_m}\right)^4 \frac{Nr^6}{\lambda^2}$	[5]
Rayleigh-Gans-Debye	rods	$\lvert\frac{n_p}{n_m} - 1\rvert \ll 1$; $r\lvert\frac{n_p}{n_s} - 1\rvert \ll \lambda$	$\left(\frac{n_p}{n_m}\right)^4 \frac{Nr^2}{\lambda^2}$	[5]
Jöbst	sph.	$\frac{2\pi r}{\lambda} \gg 1$; $r \gg \frac{\lambda}{\lambda^2}$	$\left(\frac{n_p}{n_m}\right)^4 \frac{Nr^4}{\lambda^2}$	[6]
van de Hulst (Anomalous Diffraction)	arb.	$\lvert(\frac{n_p}{n_m})^2 - 1\rvert \ll 1$; $2\pi r \gg \lambda$	$\frac{N\lambda}{r(\frac{n_p}{n_m})^2} \sin(\frac{r}{\lambda}(\frac{n_p}{n_m})^2) + \frac{N\lambda^2}{r} \frac{[1-\cos(\frac{r}{\lambda}(\frac{n_p}{n_m})^2)]}{(\frac{n_p}{n_m})^2}$	[7]
Mie	sph.	$r \gg \lambda$	$N\left[\frac{\lambda}{r^2} + \frac{\lambda}{r^2}(\frac{n_p}{n_m})^4\right]$	[6]
Hart-Montroll	sph.	$1 < (\frac{n_p}{n_m})^2 < 1.5$; $0.25\lambda < \pi r < 3\lambda$	$\frac{Nr^2}{\lambda^2} \frac{[(\frac{n_p}{n_m})^2-1]^2}{(\frac{n_p}{n_m})^2} \left[(\frac{n_p}{n_m})^8 + (\frac{n_p}{n_m})^4(\frac{\lambda}{r})^2 + (\frac{\lambda}{r})^4\right]$	[6,8]
Perelman	arb.	$\lvert(\frac{n_p}{n_m})^2 - 1\rvert \ll 1$; $2\pi r \gg \lambda$	$N\frac{n_p}{n_m}(\frac{r}{\lambda})^5((\frac{n_p}{n_m})^4 - 1)^2$	[6]
Evans-Fournier	arb.	$\lvert\frac{n_p}{n_m}\rvert < 1$; any r, λ	$N\left(\frac{r}{\lambda} + \frac{r}{\lambda}^3 + \frac{r}{\lambda}^5\right) \left[\left(\frac{\frac{r}{\lambda}+\frac{r}{\lambda}^3+\frac{r}{\lambda}^5}{\left(\frac{\lambda}{r}\left(\frac{n_m}{n_p}\right)^2 \sin\left(\frac{r}{\lambda}\left(\frac{n_p}{n_m}\right)^2\right)+\left((\frac{\lambda}{r})^2 \frac{\cos(\frac{r}{\lambda}(\frac{n_p}{n_m})^2)}{(\frac{n_p}{n_m})^2}\right)\right)}\right)^{\frac{\lambda}{r}}\right]^{-\frac{r}{\lambda}}$	[6,9]
Eikonal	arb.	$\lvert(\frac{n_p}{n_m})^2 - 1\rvert \ll 1$; $2\pi r \gg \lambda$	$N(\frac{\lambda}{r})^2\left(\frac{\cos(\arctan((\frac{n_m}{n_p})^2))}{(\frac{n_p}{n_m})^2}\right)^2 \left[\cos((\frac{n_m}{n_p})^2) - \cos\{\frac{r}{\lambda}(\frac{n_p}{n_m})^4\} - \arctan[(\frac{n_m}{n_p})^2]\exp\{-\frac{(\frac{n_p}{n_m})^2 r}{\lambda}\}\right] - \frac{N\lambda}{r(\frac{n_p}{n_m})^4}\cos[\arctan(\frac{n_m}{n_p})^2 \sin(\frac{r}{\lambda}(\frac{n_p}{n_m})^4)] - N\arctan((\frac{n_m}{n_p})^2)\exp\{-\frac{(\frac{n_p}{n_m})^4 r}{\lambda}\}$	[6,10]

Supplementary Table 2: Selection of refractive indexes collated from different literature sources. Mostly bacteria and yeast are given, but we also include some other biological samples for completeness.

**Bacteria**	**Refractive index**	**Conditions**	
E. coli	1.3324	λ =600 nm	[11]
	1.382	minimal absorbance method	[12]
	1.387	immersive refractometry	[12]
	1.388	index matching	[13]
	1.395	λ =589 nm	[14]
	1.397	λ =589 nm	[15]
	1.406 ± 0.003	λ =589 nm	[16]
E. coli DH1	1.382	λ =350 nm	[17]
E. coli cytoplasm	1.390	immersive refractometry	[12]
E. coli cytoplasm and nucleoid	1.382	immersive refractometry	[12]
E. coli nucleoid	1.371	immersive refractometry	[12]
E. coli cell wall	1.4	λ =520 nm	[18]
E. coli protoplast	1.35	λ =520 nm	[18]
Bacillus cereus	1.386	λ =520 nm	[18]
	1.3865	λ =542 nm	[19]

B. cereus var. mycoides	1.4000	λ =542 nm	[19]
B. cereus spores	1.521	λ =542 nm	[19]
B. cereus var. mycoides spores	1.528	λ =542 nm	[19]
B. megaterium	1.3880	λ =542 nm	[19]
B. megaterium spores	1.537	λ =542 nm	[19]
B. subtillus	1.446	index matching	[13]
C. oligotrophus	1.365<br>1.371	λ =350 nm<br>minimal absorbance method	[17]<br>[17]
Lactobacillus bulgaricus	1.404	λ =589 nm	[20]
Marinobacter articus	1.371	λ =350 nm	[17]
Micrococcus lysodeikticus	1.399	λ =589 nm	[16]
Proteus vulgaris	1.385	immersive refractometry	[21]
Sarcina lutea	1.396	immersive refractometry	[21]
Serratia marcescens	1.387 ± 0.001	λ =589 nm	[16]
Staphylococcus aureus	1.413 ± 0.002	λ =546 nm	[16]
Streptococcus haemolyticus	1.392	immersive refractometry	[21]
Streptococcus faecalis	1.37	immersive refractometry	[22]

**Other**			
Yeast - nucleus	1.36-1.39	λ =633 nm	[23]
Yeast - cell wall	1.53	Theoretical calculation	[24]
Lipid	1.50	approximate as oil	[25]
Mitochondria	1.40	λ =632.8 nm	[26]
Mitochondrion - resting	1.43	theoretical calculation	[27]
Mitochondrion - respiring	1.50<br>1.35	membrane and matrix<br>intramembrane	[27]<br>[27]
Microtubules	1.512	index matching	[28]
HeLa Cells	1.33-1.39<br>1.385 ± 0.001<br>1.3716 ± 0.0035	λ =633 nm<br>λ =632.8 nm<br>λ =632.8 nm	[23]<br>[29]<br>[30]
HeLa - nuclei	1.3554 ± 0.0031<br>1.3528 ± 0.0035	λ =632.8 nm<br>mechanically isolated	[30]<br>[30]
HL60 (Human myelocytic Leukemia Cells)	1.3776 ± 0.0046	λ =632.8 nm	[30]
HL60 - nuclei	1.3582 ± 0.0030	λ =632.8 nm	[30]
Jurkat (leukemic T-cells)	1.3671 ± 0.0052	λ =632.8 nm	[30]
Jurkat - nuclei	1.3610 ± 0.0037	λ =632.8 nm	[30]
MCF7 (Human breast cancer cells)	1.3713 ± 0.0048	λ =632.8 nm	[30]
MCF7 - nuclei	1.3554 ± 0.0031	λ =632.8 nm	[30]
Human plasma proteins	1.60	λ =589.3 nm	[31]
Plasma β-lipoprotein	1.514	λ =589.3 nm	[31]
Human ribonuclease	1.630	index matching	[32]
Human β-lactoglobulin	1.594	index matching	[32]
Human Pepsin	1.603	index matching	[32]

Supplementary Table 3: Refractive index of solutions containing components that may be released during growth (particularity when grown under sublethal antibiotic or any other stress concentrations) as measured by a refractometer. The maximum concentration of extracellular DNA that could be released during normal growth is estimated to be 7.45 µg ml^{-1} given by: concentration of cells × size of chromosome × mass of base pair / Avogadros constant. Assuming lysis of all cells from the maximum cell concentration observed during our experiments the concentration can be calculated as: $1.5 \times 10^9 \text{ml}^{-1} \times$ 4.6M bases $\times 650/6.02 \times 10^{23}$. The small difference between the refractive index of H_2O and extracellular bacterial DNA is negligible meaning bacterial cell lysis will not noticeably effect the calibration curve.

Solution	Refractive index
H_2O	1.335
802.8 µg ml^{-1} 23-mer primer DNA	1.335
1099.4 µg ml^{-1} 23-mer primer DNA	1.336
LB medium	1.338
Cell Lysate (grown in LB then sonicated)	1.341

Supplementary Table 4: 2nd degree polynomial fit parameters obtained using the curve fitting tools available in the Matlab environment [33]. For each sample, the equation was solved for OD = $0.05, 0.1, 0.5, 1$ and 10 and plotted in Figure 2C.

Sample	Equation	R-squared
15 µm beads	$4.21 \times 10^9\text{OD}^2 + 1.63 \times 10^{11}\text{OD} + 7.64 \times 10^9$	0.995105
10 µm beads	$4.17 \times 10^8\text{OD}^2 + 4.17 \times 10^8\text{OD} + 2.62 \times 10^8$	0.988257
3.0 µm beads	$1.80 \times 10^6\text{OD}^2 + 1.52 \times 10^7\text{OD} + 6.60 \times 10^6$	0.972179
1.0 µm beads	$8.82 \times 10^5\text{OD}^2 + 1.19 \times 10^6\text{OD} + 1.28 \times 10^6$	0.981168
0.5 µm beads	$2.05 \times 10^5\text{OD}^2 + 4.70 \times 10^5\text{OD} + 2.35 \times 10^5$	0.991838

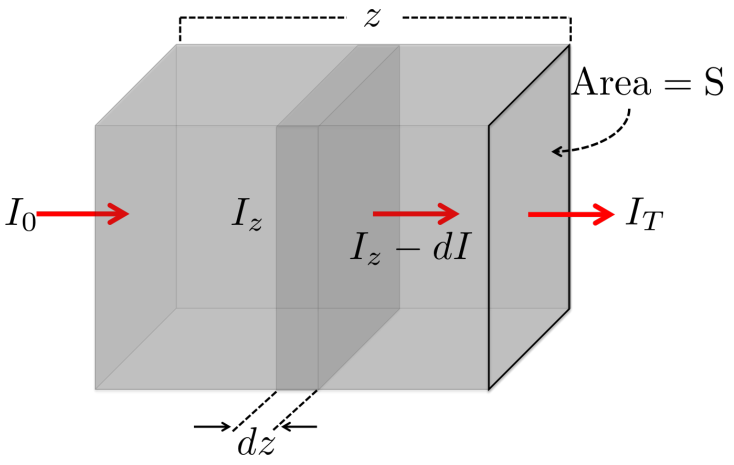

Supplementary Figure 10: Schematic outlining parameters used in the derivation of the Beer-Lambert Law (BLL). A beam of intensity I_0 enters the sample perpendicular to area, S. As the sample passes through a thin slice of the sample (dz) the intensity has reduced from I_z to $I_z - dz$ and having passed through the length of the sample (z) the intensity has reduced to the transmitted intensity (I_T).

Supplementary Note 1: Beer-Lambert law derivation

Consider a beam of parallel, monochromatic light with intensity I_0 striking a sample of area, S perpendicular to the surface (Supplementary Fig. 10). The light travels through the sample to a depth of z, after which the intensity is reduced to I_T. An infinitesimally thin slice of the sample, dz contains M molecules, such that

$$M = CSdz \tag{1}$$

where C is the number of molecules per unit volume. Each molecule has a cross-section σ meaning that the fraction of light absorbed due to a single molecule of area s is $\frac{\sigma}{s}$. Therefore, the total cross-sectional area from all the molecules in the block is given by

$$CSdz\frac{\sigma}{s} = C\sigma dz \tag{2}$$

If we compare the intensity of light entering this thin slice (I_z) with the intensity of light that exits ($I_z - dI_z$) we find the fraction of absorbed light I_a is

$$I_a = \frac{dI_z}{I_z} \tag{3}$$

The sum of I_a through all the slices of the sample is proportional to the sum of all cross-sectional areas for all molecules in all the thin slices (Eq. 2). Integrating Eq. 3 we have:

$$\int_{I_0}^{I_T} \frac{dI_z}{I_z} = -\int_0^Z C\sigma dz \tag{4}$$

which can be rewritten in its common form as the Beer-Lambert law (BLL)

$$\ln\left(\frac{I_0}{I_T}\right) = C\sigma Z \tag{5}$$

When BLL is applied to OD measurements σ is the cross-section of the bacterium; z the distance the light travels through the sample, I_0, the intensity of light incident on the sample and I_T is the intensity of light transmitted through the sample and related to the OD through the expression:

$$\text{OD} = C\sigma Z - \log(I_0) \tag{6}$$

Supplementary Note 2: Multiple Scattering considerations

As the concentration of bacteria increases, the possibility of multiple scattering, where light is scattered off more than one bacterium, increases due to the closer packing of the bacteria within the sample (Figure 1B, main text). Subsequently, these multiple scattering events start to contribute significantly to the measured intensity of light at the detector.

For particles larger or comparable to the wavelength of radiation a correction factor for multiple scattering events can be added to the BLL using the small-angle approximation. This takes the form of correction factor CF [34–41]

$$CF(z,\sigma) = \sum_{m=1}^{\infty} \frac{(\frac{\sqrt{2}}{w_0}z)^m}{m!} \frac{1 - S(m)}{1 - \exp\left[-\frac{w_0 r}{\sqrt{2}} r_d^2\right]} \tag{7}$$

where m is the number of scattering events the light has undergone and $m_{max} = \infty$, r the radius of the scatterer, w_0 the beam width of incident light, z the distance between the light source and detector, r_d is the distance between the two scattering events and $S(m)$ is the integral over all particles and scattering events that occur before the light enters the detector and is defined as

$$S(m) = \int_0^1 \dots \int_0^1 ds_1 \dots ds_m \exp\left[\frac{-\frac{\sqrt{2}}{w_0} r_d^2}{1 + \frac{\frac{\sqrt{2}}{w_0}z^2}{\alpha} \sum_{i=1}^m s_i^2}\right] \tag{8}$$

where α is the polarisation of the scattered light and s_m is the number of scatterers the light interacts with [34–41]. CF increases the power measured at the detector when multiple scattering occurs.

The inclusion of the above corrections allows calculation of the effect multiple scattering events have on the intensity of light on the detector (I_D), but with limitations and at a cost.

$CF(z, \sigma)$ is nonlinear with N, since CF is obtained by integrating over the area of each individual scatterer (s_m), an increase in N will lead to a reduction in r_d and increased probability of m increasing. Initially, as N increases $CF(z, \sigma)$ also increases. But, for multiple scattering events where a single light beam is scattered many times, $CF(z, \sigma) \to 0$. In this regime N is so large that all light entering the sample is constrained by photon diffusion and no light is directly transmitted to the detector. Consequently, $I_D \to 0$ meaning that OD becomes very large. In the case of low density samples, CF reduces to single scattering theory and the BLL holds. In the multiple scattering regime CF is significant and has been shown to increase I_D by 50 % for particulates in fog at the small value of $m = 2$. [41]

However, in order to apply the correction factor to OD measurements it is necessary for the user to know both m and N. Thus, in order to measure N accurately using a spectrophotometer, N needs to be accurately determined by another method. Furthermore, since the forward scattering contribution to the power incident on the detector is highly dependent upon the geometry of the scatterer as well as the density and absorption; the degrees of freedom within the system require calibration of the OD vs cell number measurements independently.

Other considerations

In the above calculations we have made two assumptions that should be noted, the sample is static over the time period of a measurement and the sample is not heterogenous in geometry.

Static vs Dynamic

Unlike dust particles, crystal structures, water droplets or colloidal solutions, for which many of these approximations were originally derived, bacteria are motile. Dynamic samples can add additional complexity to solutions of scattering (see [42] for a recent discussion or [43] for a more rigorous review). Often, OD measurements are conducted in rich growth media (LB or defined rich media like EZ Rich [44]) where samples are not nutrient limited leading to reduced production of flagella and therefore limited motility. In this case it is safe to assume that samples undergo only diffusion during measurement of I_D. However, if the growth conditions deviate from optimal, the bacteria become motile [45]. To estimate the effect on motility we take the case of *E. coli* and previously reported swimming speed of 20 µm/ sec [46]. At this speed *E. coli* will only have traveled 0.2 µm (or less than half the body length) in the time it takes a 50 Hz platereader to acquire a single OD measurement, and we can still assume a static sample. However, the effect of swimming should be re-evaluated for cases where it might play a more significant role, for example smaller and faster bacteria.

Heterogeneity of the geometry

The effect of heterogeneity of a sample can be discounted for samples with dimensions $D \gg \lambda$ (e.g. yeast), however we have shown that it is more important in bacterial cultures where $D \approx \lambda$. In conditions where heterogeneity in bacterial geometry does not change throughout the growth cycle of the culture it is sufficient to perform one calibration curve (Fig. 3). But, in cases where the heterogeneity of the sample does change, even if the bacteria sizes do not (for example bigger and smaller bacteria are present where the ratio of the two changes through the growth cycle), OD is no longer suitable for estimated of bacterial number density and direct counting should be performed.

Volume of sample in the well

There is a positive correlation between the volume of sample through which light travels and the probability of multiple scattering events occurring that is not necessarily in accordance with the BLL. In order to determine the effect of differences in the volume size on the calibration, we measured OD

for solutions of 1 µm diameter beads at known dilutions from manufacturer concentration 1.8×10^{11} ml^{-1} for 100 µl, 200 µl and 300 µl volumes of solution in a plate reader well (Supplementary Fig. 9).

The maximum volume of solution (300 µl) corresponds to the maximum volume the plate reader well can hold ($l = z$), meaning the smaller volumes correspond to $z/3$ and $2z/3$ respectively. The BMG Spectrostar spectrophotometer performs path-length correction to the measurements by applying the BLL approximation. For cases where the BLL is satisfied, the measured calibration curves for varying l would collapse to a single line. Supplementary Figure 9 shows that, unlike for absorbance measurements, for turbidity measurements this is not the case. Instead, variation in l leads to significant deviation between the three lines as the concentration of the solution increases. Deviation at high concentrations is as expected, with OD saturating quickest for $l = z$. At low fractions of manufacturer concentration, the three curves still do not fully collapse to a single line (inset) with OD being higher for $l = z$ compared to $l = \frac{z}{3}$ and $l = \frac{2z}{3}$. While this effect is pronounced, for most plate reader experiments, it can be neglected provided the volume of the sample in each well is kept the same and that the volume does not change significantly throughout the duration of the experiment (e.g. evaporation of the solution is prevented or minimized).

OD at transitions between different scattering regimes

To best determine the limits of the single, multiple and photon diffusion scattering regimes, it is useful to consider the fraction of the total volume of the culture occupied by scatterers (ρ) rather than C, and several previous studies have evaluated the applicability of different scattering theories with respect to ρ [47–50]. From these, single scattering theory is applicable for volume fractions of $\rho \leq 0.001$ [49,50], the multiple scattering regime is satisfied for $0.001 \leq \rho \leq 0.2$ [47,49] and for higher values ($\rho > 0.2$), photon diffusion limit is reached where statistical approaches are best suited [47–50].

References

[1] A. Hecht, D. Endy, M. Salit, and M. S Munson. When wavelengths collide: Bias in cell abundance measurements due to expressed fluorescent proteins. *ACS Synth Biol*, 5:1024–1027.

[2] Albert L Koch. Some Calculations on the Turbidity of Mitochondria and Bacteria. *Biochimica et biophysica acta*, 51:429–441, 1961.

[3] P. S. Swain, K. Stevenson, A. Leary, L. F. Montano-Gutierrez, I. B. N. Clark, J. Vogel, and T Pilizota. Inferring time-derivatives, including cell growth rates, using gaussian processes. *BioRxiv*, 2016.

[4] Akira Ishimaru and Yasuo Kuga. Attenuation constant of a coherent field in a dense distribution of particles. *Journal of the Optical Society of America*, 72(10):1317, 1982.

[5] Lord Rayleigh. On the scattering of light by small particles. *Philosophical Magazine*, 41(4):447–454, 1871.

[6] Subodh K. Sharma and David J. Sommerford. *Light scattering by optically soft particles*, volume 1 of *Springer Praxis Books*. Springer Berlin Heidelberg, Cambridge, 2006.

[7] H C Van De Hulst. *Light scattering by small particles*, volume 1. Dover Publications, New York, 1981.

[8] S. K. Sharma and D. J. Somerford. Relationship between the S approximation and the Hart-Montroll approximation. *Journal of the Optical Society of America A*, 13(6):1285, 1996.

[9] B T Evans and G R Fournier. Simple approximation to extinction efficiency valid over all size parameters. *Applied optics*, 29(31):4666–4670, 1990.

[10] S.K. Sharma and D.J. Somerford. *Scattering of Light in the Eikonal Approximation*, volume 39 of *Progress in Optics*. Elsevier, London, 1999.

[11] Aleksei E. Balaev, K. N. Dvoretski, and Valeri A. Doubrovski. Refractive index of *Escherichia coli* cells. In *SPIE 4707, Saratov Fall Meeting 2001: Optical Technologies in Biophysics and Medicine III*, pages 253–260, 2002.

[12] J. A C Valkenburg and C. L. Woldringh. Phase separation between nucleoid and cytoplasm in *Escherichia coli* as defined by immersive refractometry. *Journal of Bacteriology*, 160(3):1151–1157, 1984.

[13] P. Y. Liu, L. K. Chin, W. Ser, T. C. Ayi, P. H. Yap, T. Bourouina, and Y. Leprince-Wang. Real-Time measurement of single bacterium's refractive index using optofluidic immersion refractometry. *Procedia Engineering*, 87:356–359, 2014.

[14] F D Bryant, B a Seiber, and P Latimer. Absolute optical cross sections of cells and chloroplasts. *Archives of biochemistry and biophysics*, 135(1):97–108, 1969.

[15] Aleksei E. Balaev, Konstanten N. Dvoretski, and Valeri A. Doubrovski. Determination of refractive index of rod-shaped bacteria from spectral extinction measurements. In Valery V. Tuchin, editor, *Saratov Fall Meeting 2002: Optical Technologies in Biophysics and Medicine IV*, pages 375–380, 2003.

[16] J. B. Bateman, Jack Wagman, and E. L. Carstensen. Refraction and absorption of light in bacterial suspensions. *Kolloid-Zeitschrift & Zeitschrift für Polymere*, 208(1):44–58, 1966.

[17] B. R. Robertson, D. K. Button, and A. L. Koch. Determination of the biomasses of small bacteria at low concentrations in a mixture of species with forward light scatter measurements by flow cytometry. *Applied and Environmental Microbiology*, 64(10):3900–3909, 1998.

[18] P J Wyatt. Differential light scattering: A physical method for identifying living bacterial cells. *Applied Optics*, 7(10):1879–1896, 1968.

[19] K F Ross and E Billing. The water and solid content of living bacterial spores and vegetative cells as indicated by refractive index measurements. *Journal of General Microbiology*, 16(2):418–25, 1957.

[20] K F A Ross. The size of living bacteria. *Quarterly Journal of Microscopical Science*, s3-98:435–454, 1957.

[21] R. Barer, K. F. A. Ross, and S. Tkaczyk. Refractometry of living cells. *Nature*, 171(4356):720–724, 1953.

[22] L. Daneo-Moore, D. Dicker, and M. L. Higgins. Structure of the nucleoid in cells of *Streptococcus faecalis*. *Journal of Bacteriology*, 141(2):928–937, 1980.

[23] Wonshik Choi, Christopher Fang-Yen, Seungeun Oh, Niyom Lue, Ramachandra R Dasari, Michael S. Feld, and Kamran Badizadegan. Tomographic phase microscopy. Quantitative imaging of living cells. *BIOforum Europe*, 10(c):2–3, 2007.

[24] R.D. Chaudhari, J.D. Stenson, T.W. Overton, and C.R. Thomas. Effect of bud scars on the mechanical properties of *Saccharomyces cerevisiae* cell walls. *Chemical Engineering Science*, 84:188–196, 2012.

[25] L Sacconi, I M Tolic-Nørrelykke, M D'Amico, F Vanzi, M Olivotto, R Antolini, and F S Pavone. Cell imaging and manipulation by nonlinear optical microscopy. *Cell biochemistry and biophysics*, 45(3):289–302, 2006.

[26] Michael Taylor. *Quantum microscopy of biological systems*. Springer Theses. Springer International Publishing, Cham, 2015.

[27] Roland Thar and Michael Kühl. Propagation of electromagnetic radiation in mitochondria? *Journal of Theoretical Biology*, 230(2):261–270, 2004.

[28] Hidemi Sato, Gordon W Ellis, and Shinya Inoui. Microtubular origin of mitotic spindle form birefringence. Demonstration of the Applicability of Wiener ' s Equation. *The Journal of Cell Biology*, 67:501–517, 1975.

[29] J Beuthan, O Minet, J Helfmann, M Herrig, and G Müller. The spatial variation of the refractive index in biological cells. *Physics in Medicine and Biology*, 41(3):369–382, 1996.

[30] Mirjam Schürmann, Jana Scholze, Paul Müller, Jochen Guck, and Chii J. Chan. Cell nuclei have lower refractive index and mass density than cytoplasm. *Journal of Biophotonics*, 9:1–9, 2016.

[31] S. H. Armstrong, M. J. E. Budka, K. C. Morrison, and M Hasson. Preparation and properties of serum and plasma proteins. XII. The refractive properties of the proteins of human plasma and certain purified fractions 1,2. *Journal of the American Chemical Society*, 69(7):1747–1753, 1947.

[32] Thomas L. McMeekin, Mildred Wilensky, and Merton L. Groves. Refractive indices of proteins in relation to amino acid composition and specific volume. *Biochemical and Biophysical Research Communications*, 7(2):151–156, 1962.

[33] The MathWorks Inc. Matlab and statistics toolbox release, MATLAB version 7.10.0 (R2010a). 2010.

[34] Andrew Zardecki. Multiple scattering corrections to the Beer-Lambert law. In John C. Leader, editor, *SPIE 0410, Laser Beam Propagation in the Atmosphere*, pages 103–110, Arlington, 1983.

[35] A. Zardecki and W G Tam. Multiple scattering corrections to the Beer-Lambert law. 2: Detector with a variable field of view. *Applied Optics*, 21(13):2413–20, 1982.

[36] A. Zardecki and W G Tam. Iterative method for treating multiple scattering in fogs. *Canadian Journal of Physics*, 57(9):1301–1308, 1979.

[37] Andrew Zardecki and Siegfried A W Gerstl. Multi-Gaussian phase function model for off-axis laser beam scattering. *Applied Optics*, 26(15):3000, 1987.

[38] W G Tam and A Zardecki. Multiple scattering of a laser beam by radiational and advective fogs. *Opt. Acta*, 26(5):659–670, 1979.

[39] W. G. Tam and A. Zardecki. Laser beam propagation in particulate media. *Journal of the Optical Society of America*, 69(1):68, 1979.

[40] W. G. Tam. Multiple scattering corrections for atmospheric aerosol extinction measurements. *Applied Optics*, 19(13):2090, 1980.

[41] W. G. Tam and Andrew Zardecki. Multiple scattering corrections to the Beer-Lambert law 1: Open detector. *Applied Optics*, 21(13):2405, 1982.

[42] Achim M. Loske, Elba M. Tello, Susana Vargas, and Rogelio Rodriguez. *Escherichia coli* viability determination using dynamic light scattering: A comparison with standard methods. *Archives of Microbiology*, 196(8):557–563, 2014.

[43] S E Harding. Applications of light scattering in microbiology. *Biotechnology and Applied Biochemistry*, 8(6):489–509, 1986.

[44] Frederick C Neidhardt, Philip L Bloch, and David F Smith. Culture medium for enterobacteria. *J Bacteriol*, 119(3):736–747, 1974.

[45] J Adler and Bonnie Templeton. The effect of environmental conditions on the motility of *Escherichia coli*. *Journal of General Microbiology*, 46(2):175–184, 1967.

[46] Jana Schwarz-Linek, Jochen Arlt, Alys Jepson, Angela Dawson, Teun Vissers, Dario Miroli, Teuta Pilizota, Vincent A. Martinez, and Wilson C K Poon. *Escherichia coli* as a model active colloid: A practical introduction. *Colloids and Surfaces B: Biointerfaces*, 137:2–16, 2016.

[47] Giovanni Zaccanti, Samuele Del Bianco, and Fabrizio Martelli. Measurements of optical properties of high-density media. *Applied Optics*, 42(19):4023–4030, 2003.

[48] G Göbel, J Kuhn, and J Fricke. Dependent scattering effects in latex-sphere suspensions and scattering powders. *Waves in Random Media*, 5(4):413–426, 1995.

[49] Akira Ishimaru. *Wave Propagation and Scattering in Random Media.* IEEE, 1999.

[50] Arianna Giusto, Rosalba Saija, Maria Antonia Iatì, Paolo Denti, Ferdinando Borghese, and Orazio I Sindoni. Optical properties of high-density dispersions of particles: application to intralipid solutions. *Applied Optics*, 42(21):4375–4380, 2003.
